# Vendor Hygiene Practices, Temporal Variation, and Microbial Quality of Soya Kebabs Sold in Public and Private Basic Schools in Sunyani, Ghana

**DOI:** 10.1002/fsn3.71909

**Published:** 2026-05-23

**Authors:** Afia Sakyiwaa Amponsah, Barikisu Mohammed, Moses Golly, Emmanuel Tetteh Doku, Belinda Agyei‐Poku

**Affiliations:** ^1^ Department of Hospitality and Tourism Sunyani Technical University Sunyani Ghana; ^2^ Department of Pharmaceutical Sciences Sunyani Technical University Sunyani Ghana

**Keywords:** basic schools, food safety, Ghana, microbial contamination, soya kebabs, vendor hygiene

## Abstract

Food safety in basic schools across developing countries poses significant public health challenges. Soya kebabs, popular protein‐rich snacks sold in Ghanaian schools, have received limited microbiological safety assessment despite widespread consumption. This study investigated the microbial quality of soya kebabs sold in basic schools (covering primary and junior high education, grades 1–9) within Sunyani Municipality, Ghana, examining relationships between vendor hygiene practices and contamination levels. A cross‐sectional study was conducted across 25 basic schools from March to June 2025. Soya kebab samples (*n* = 50) were collected from 25 vendors at two time points (early week: Monday–Tuesday; late week: Thursday–Friday) and analyzed for total aerobic counts, coliforms, Enterobacteriaceae, *Escherichia coli* , *Staphylococcus aureus* , and fungi using standard microbiological methods. Vendor hygiene assessments were performed using structured observation checklists for all 25 vendors. Bacterial contamination levels varied substantially throughout the school week. Total aerobic counts ranged from 4.18 ± 0.13 to 4.71 ± 0.20 log_10_ CFU/g, while *S. aureus* contamination ranged from 2.28 ± 0.13 to 4.12 ± 0.10 log_10_ CFU/g (*p* < 0.001). *E. coli* levels ranged between 1.85 ± 0.16 and 2.43 ± 0.19 log_10_ CFU/g. Public schools showed significantly higher contamination than private schools across multiple parameters. Vendor assessments revealed critical deficiencies: only 16% demonstrated adequate hand washing, 28% had hand washing facilities, and 12% maintained proper temperature control. A strong negative correlation (*r* = −0.780, *p* < 0.001) existed between hygiene scores and contamination levels. The study reveals substantial quality variation in soya kebabs throughout the school week and widespread hygiene deficiencies posing significant health risks to students. Targeted interventions addressing vendor training and infrastructure provision could substantially reduce these food safety risks.

## Graphical abstract

This study evaluated microbial contamination and vendor hygiene practices associated with soya kebabs sold in public and private basic schools in Sunyani Municipality, Ghana. Significant increases in bacterial contamination were observed throughout the school week, with poor hygiene practices strongly associated with elevated contamination levels. Public schools exhibited higher levels of contamination than private schools, highlighting the need for targeted food safety interventions and vendor training.

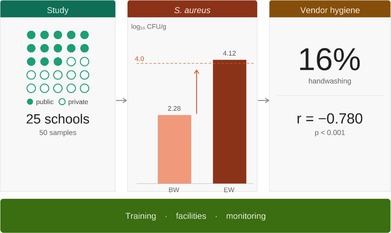

## 1 Introduction

Food safety in educational settings has become a global public health priority, with sustainable approaches requiring data‐driven risk management strategies (Wiedmann 2025). The framework for determining and understanding risks associated with food contamination and safety in the food sector is multifaceted and includes hygienic practices, environmental sanitation factors, and pathogen characterization (WHO 2025). Food safety in developing countries is particularly concerning due to poor enforcement of hygienic practices and sanitation laws (Christiana Cudjoe et al. 2022; Silvanus et al. 2025) compounded by limited regulatory oversight and inadequate food control systems (Grace 2023; Tibebu et al. 2024). Children under 5 years of age carry 40% of the global foodborne disease burden, with 125,000 deaths occurring annually (WHO 2024).

Predictive microbiology research has established that foodborne pathogen levels exceeding 4 log_10_ CFU/g pose significant infection risks to children, utilizing mathematical models to forecast microbial behavior and enable accurate risk assessments (FDA 2024). This threshold is particularly critical for vulnerable populations in school settings where collective catering systems serve large numbers of children (Taiwo et al. 2024).

In developing countries, school food systems face several challenges, including balancing accessibility, nutritional adequacy, and safety. The lack of potable water and electricity, and inadequate infrastructure, can complicate or negate oversight of food production and routine monitoring of foodborne hazards (Subedi et al. 2025). The development and adoption of food safety systems are very inconsistent among developing countries, with many lacking the regulatory capacity to ensure comprehensive oversight (Henson and Jaffee 2008).

The Ghana School Feeding Programme (GSFP) serves over 2.6 million children but often falls short of recommended nutrient levels. Previous assessments have revealed hygiene deficiencies in handwashing practices and food preparation environments (Annan et al. 2022; Bigson et al. 2020). Informal food vendors supplement formal programs by selling a variety of foods, including soya kebabs, directly to schoolchildren (Fernandes et al. 2017; Ogum‐Alangea et al. 2020). However, regulatory enforcement and infrastructure provision remain inadequate (Ahiabor et al. 2024).

Soybean‐derived products, particularly soya kebabs (processed from soybean curd), are a protein source in school nutrition programs. Soybeans ( *Glycine max* L.) provide a high biological value protein source, and they are also high in lipids and oligosaccharides, requiring substantial microbial enzymatic activities, including proteases, lipases, and amylases for proper degradation during processing (Ahnan‐Winarno et al. 2021).

In school environments, soya kebabs are typically sold cold in plastic containers and packaged in polyethene bags, sometimes with the addition of spicy pepper powder (*yaaji*) upon purchase. Previous microbial assessments of meat kebabs in tertiary schools showed levels exceeding acceptable limits, thus the need for enhanced regulatory oversight (Azumah et al. 2021). Popular pathogenic microbes in Ghana include bacteria and fungi, with particular concern for *Staphylococcus aureus* , *Salmonella* spp., and fecal coliforms in street‐vended foods (Ahiabor et al. 2024). Although affordable meals from informal vendors are essential for improving school attendance and retention, supporting long‐term goals to address poverty and educational challenges in Ghana, the imperative for high‐quality, safe food cannot be compromised. The reliance on informal food sources may perpetuate health risks, thus the need for a balance between accessibility and safety, particularly given that over 90% of the foodborne disease burden falls on people in low‐ and middle‐income countries (Grace 2023).

Sunyani Municipality serves as the administrative and commercial capital of Ghana's Bono Region, representing a typical mid‐sized transitional city with a population where informal food vendors serve significant student populations across numerous educational institutions (Anaafo et al. 2024). In general, basic schools rely heavily on these vendors to provide affordable nutrition options for students (Ogum‐Alangea et al. 2020). Assessment of food vendors in the broader Brong Ahafo region (now Bono Region) revealed variable compliance with food hygiene practices (Akparibo et al. 2021). Previous food safety studies in the area have documented antibiotic residues in meat products and various contamination issues among street food vendors, highlighting systemic challenges in the informal food sector (Akansale et al. 2019).

Despite various studies on foodborne microbiological hazards in Ghana, no research has examined the microbial quality of soya kebabs sold in basic schools (covering primary and junior high education, grades 1–9 in the Ghanaian education system), despite their widespread consumption among school‐aged children (Ahiabor et al. 2024). This study addresses this knowledge gap by investigating the microbial quality of soya kebabs in Sunyani Municipality basic schools, examining the relationship between vendor hygiene practices and contamination levels, and comparing food safety outcomes between public and private schools. These findings directly inform interventions targeting the informal food sector serving vulnerable school populations.

## 2 Materials and Methods

### 2.1 Study Design and Area

This cross‐sectional analytical study was carried out in the Sunyani Municipality, Bono Region, Ghana, between March and June 2025. The study used a stratified sampling design, collecting samples at two time points each week (early‐week and late‐week) to assess temporal changes in contamination.

### 2.2 Ethical Considerations

This study was approved by the Sunyani Technical University Research Ethics Committee (Approval No.: STU/REC/2025/003, approved February 15, 2025). The study protocol, informed consent forms, and data collection instruments were reviewed and approved prior to research commencement.

Written informed consent was obtained from school authorities (headteachers or designated administrators) at each participating school prior to conducting any research activities on school premises. Permission letters clearly outlined the study purpose, procedures, expected duration of activities, and confidentiality protections.

Verbal informed consent was obtained from each vendor participant after clearly explaining in their preferred language (Twi or English): (1) the study purpose and procedures, (2) that participation was entirely voluntary with no consequences for declining, (3) their right to withdraw at any time, (4) confidentiality protections for their identity and business information, and (5) how findings would be used to improve school food safety. Verbal rather than written consent was used for vendors due to varying literacy levels and to reduce potential intimidation associated with formal documentation. All consent discussions were conducted by trained research assistants, and vendor consent was documented on standardized consent log forms recording date, time, and research assistant's initials, without including vendor identifying information.

Vendors were not informed they were being observed until after the observation period concluded to minimize the Hawthorne effect, the tendency for individuals to modify behavior when aware of being observed. This approach was ethically approved and ensured that observed hygiene practices reflected typical rather than improved behavior. Following observation, vendors were thanked for their participation and informed of their anonymous contribution to improving school food safety. Three vendors (5.7% of those approached) declined participation after being informed and were excluded from analysis.

Food samples were purchased from vendors at normal retail prices, identical to regular customer transactions, ensuring no disruption to vendor operations. All participants (vendors and schools) were assigned anonymous codes, with no identifying information recorded in the analysis dataset. Schools were coded by type and size only (e.g., “Public‐Large”), and vendors by sequential numbers (V001–V025).

The study posed minimal risk to participants, involving only observational assessment of publicly visible hygiene practices and microbiological analysis of food products already being sold to the public. No clinical procedures, biological sample collection from humans, or invasive assessments were conducted.

### 2.3 School Selection and Sampling Strategy

#### 2.3.1 Sample Size Calculation

The sample size was determined using the formula: *n* = (*Z*_1−*α*/2_ + *Z*_1−*β*_)^2^ × 2*σ* ^2^/*d* ^2^, where *Z*_1−*α*/2_ = 1.96 (95% confidence level), *Z*_1−*β*_ = 0.84 (80% power), *σ* = 0.4 log_10_ CFU/g (expected standard deviation based on pilot study), and *d* = 0.5 log_10_ CFU/g (minimum detectable difference). The calculated sample size: *n* = 22 per group, increased to 25 per group (50 total) to account for a 10% potential loss.

#### 2.3.2 School Selection Strategy

A two‐stage stratified random sampling method was used: Stage 1 involved dividing schools by type (public vs. private) and size (enrolment > 200 vs. < 200 students). Stage 2 involved randomly selecting schools from these groups using a computer‐generated random number generator. Inclusion criteria: basic schools with active soya kebab vendors operating at least 3 days a week within a 200 m radius. Exclusion criteria: schools with fewer than 50 students, temporary vendor operations, or refusal to participate. Selection bias was reduced by systematically replacing non‐participating schools with randomly chosen alternatives from the same group.

### 2.4 Sample Collection Protocol

#### 2.4.1 Sampling Design Clarification

The study employed a paired sampling design wherein the same 25 vendors were sampled at two distinct time points during the school week. Specifically, each vendor was sampled once during the early week period (Monday–Tuesday) and again during the late week period (Thursday–Friday), yielding 50 total samples (25 vendors × 2 time points = 50 samples). This paired design enabled within‐vendor temporal comparisons to assess whether contamination levels changed as the school week progressed, while controlling for vendor‐specific baseline characteristics (hygiene practices, equipment, training, etc.).

Vendor identification codes were maintained across both sampling time points (e.g., Vendor V001 sampled on Monday and again on Thursday, Vendor V002 sampled on Tuesday and again on Friday, etc.), ensuring that temporal changes could be attributed to weekly progression rather than differences between vendors. The two‐day window for each time point (Monday–Tuesday for “beginning of week”; Thursday–Friday for “end of week”) provided flexibility in scheduling while maintaining consistency in the temporal interval between sample collections (approximately 3–4 days between paired samples from the same vendor).

At each participating school, two samples of approximately 25 g each were collected into sterile containers directly from the vendor using aseptic techniques. Sample collection adhered to strict protocols: sterile forceps were used for handling, samples were placed in presterilized containers with secure lids, and collection time, temperature, and environmental conditions were recorded for each sample. Samples reflected typical serving portions as sold to students, to accurately capture the actual exposure conditions. Each sample was assigned a unique identifier incorporating the school code, vendor identification, collection date, time, and environmental temperature. Immediately after collection, samples were placed in insulated containers with ice packs to keep temperatures below 4°C (verified with calibrated thermometers) during transportation to the laboratory. All samples were processed within 2 h of collection to minimize the risk of post‐collection microbial growth.

### 2.5 Sample Preparation

All laboratory procedures followed ISO 17025 standards. Media were purchased from certified suppliers and tested for sterility using ATCC reference strains ( *Escherichia coli* ATCC 25922, *S. aureus* ATCC 25923, *C. albicans* ATCC 10231), with appropriate controls included in each batch. Detailed quality assurance protocols are provided in Tables S1–, S5.

Each 25 g soya kebab sample was aseptically transferred to a sterile stomacher bag containing 250 mL of 0.1% sterile peptone water (Oxoid Ltd., Hampshire, UK), providing a 1:10 dilution ratio. This dilution volume (slightly higher than the ISO‐recommended 225 mL) was selected to ensure complete sample coverage and facilitate thorough mixing during stomaching, particularly important given the heterogeneous texture of soya kebabs with varying particle sizes. Samples were homogenized using a laboratory stomacher (Seward Stomacher 400, UK) for 2 min at 260 rpm to ensure uniform distribution of microorganisms throughout the sample matrix.

### 2.6 Microbiological Parameters

#### 2.6.1 Total Aerobic Count

The enumeration of total aerobic bacteria was performed using Plate Count Agar (Oxoid Ltd., CM0325, UK) as the growth medium (Paulsen et al. 2006). Media were prepared according to the manufacturer's instructions, autoclaved at 121°C for 15 min, and cooled to 45°C ± 2°C before pouring. Aliquots of 0.1 mL from appropriate dilutions were spread onto duplicate agar plates using sterile bent glass rods. Plates were incubated at 37°C for 24–48 h, after which visible colonies were counted on a Reichert Quebec Colony Counter (Fisher Scientific, UK) and recorded.

#### 2.6.2 Microbial Isolation and Enumeration

##### 2.6.2.1 *E. coli* Detection and Confirmation

Specific detection of *E. coli* was performed using Brilliant *E. coli* selective agar (Oxoid Ltd., CM1046, UK) supplemented with TTC supplement. Sample dilutions were spread onto this chromogenic medium and incubated at 37°C for 24 h. Presumptive *E. coli* colonies (blue to purple) were confirmed through tests such as the indole production test, β‐glucuronidase activity, Gram staining, and microscopy. Confirmed isolates were stored at −80°C in glycerol broth.

##### 2.6.2.2 *S. aureus* Enumeration and Confirmation

Mannitol Salt Agar (Oxoid Ltd., CM0085, UK) was employed for selective enumeration. Yellow colonies indicative of mannitol fermentation were confirmed as *S. aureus* based on the following tests: Catalase test (positive), Coagulase test using rabbit plasma, and Protein A detection with the latex agglutination kit (Oxoid Ltd.). Confirmed *S. aureus* isolates were subsequently tested for enterotoxin production capability using ELISA (TECRA Staph Enterotoxin Kit).

#### 2.6.3 Coliform Enumeration

Coliform bacteria enumeration was performed using MacConkey agar (Oxoid, CM0115B, UK) (Hyera 2015). The spread plate technique was used, with 0.1 mL of the sample dilutions spread onto duplicate plates. Initial incubation occurred at 37°C for 24 h to enumerate total coliforms. A parallel set of plates was incubated at 45°C for 24 h to count thermotolerant coliforms, which indicates fecal contamination specifically.

#### 2.6.4 Enterobacteriaceae Enumeration

The Enterobacteriaceae family was enumerated using Violet Red Bile Glucose Agar (Oxoid, CM0485, UK) as described by Nikita (2021). Sample dilutions were plated using the spread plate method, and plates were incubated at 37°C for 24 h. Purple‐colored colonies characteristic of this bacterial family were counted and recorded, providing information about potential enteric pathogen contamination.

##### 2.6.4.1 *Salmonella* Detection

*Salmonella* detection followed a multistep enrichment protocol (Bruini 2017). Samples were initially pre‐enriched in 2% peptone solution, followed by selective enrichment in Rappaport‐Vassiliadis Broth. Enriched cultures were then streaked onto xylose lysine deoxycholate agar and incubated at 37°C for 24 h. Presumptive *Salmonella* colonies, characterized by yellow colouration with black centers indicating hydrogen sulfide production, were further confirmed through biochemical testing.

#### 2.6.5 Fungal Analysis

Yeast and mold enumeration was performed using Potato Dextrose Agar (Oxoid, CM0139B, UK) acidified to pH 3.5 to inhibit bacterial growth (Acharya and Hare 2022). Sample dilutions were spread onto duplicate plates and incubated at 25°C for up to 5 days. Yeast and mold colonies were identified based on morphological characteristics and counted separately. Representative isolates were selected for further identification based on macro and microscopic morphology.

### 2.7 Vendor Assessment

Hygiene assessments were conducted for all 25 vendors included in the study, with each vendor observed once during the initial sampling visit. Structured observations were performed using a validated 50‐point hygiene checklist developed in accordance with WHO guidelines and validated through expert consensus (*n* = 5 food safety experts; content validity index = 0.92). To ensure consistency and minimize observer bias, all hygiene observations were conducted by a team of three trained observers who completed a structured 16‐h pre‐study calibration programs covering checklist item interpretation, scoring criteria, fieldwork protocols, and strategies for minimizing perceptual bias. Prior to data collection, all observers independently scored practice observations and reconciled discrepancies through structured group discussion until consensus was reached, ensuring harmonized interpretation of checklist items across the team. During fieldwork, each vendor was assessed simultaneously and independently by two observers without conferring; where individual item scores differed by more than one point, a third independent review was conducted and consensus established. Observers were unaware of laboratory results throughout data collection, and all vendors were assigned anonymous codes to prevent prior impressions from influencing scores. Inter‐rater reliability, established across a randomly selected subset of vendors (*n* = 10), yielded Cohen's *κ* = 0.85, indicating near‐perfect agreement. The assessment covered five domains: (1) Personal hygiene (15 points), (2) food handling practices (15 points), (3) equipment and environment (10 points), (4) food safety knowledge (5 points), and (5) Infrastructure availability (5 points).

Observations were conducted during peak serving times (10:00–11:00 a.m.) to capture typical operating conditions. Each vendor was observed for a minimum of 30 min, with practices scored as follows: Adequate (2 points), partially adequate (1 point), or inadequate (0 points). Overall hygiene scores were calculated as percentages and categorized as follows: excellent (90%–100%), good (70%–89%), fair (50%–69%), poor (30%–49%), or very poor (< 30%).

Vendor observations were conducted unobtrusively during normal business hours to capture typical practices. Vendors were informed that research was being conducted on school food safety in the municipality but were not told their specific establishment was being observed until after the observation period concluded to avoid the Hawthorne effect.

### 2.8 Statistical Analysis

All analyses were conducted using R version 4.3.0 and SPSS version 25.0 at a 95% confidence interval. Data normality was assessed using the Shapiro–Wilk test. Microbial counts were log_10_ transformed to achieve normal distribution.

#### 2.8.1 Temporal and School‐Type Comparisons

For temporal comparisons (beginning vs. end of week), paired *t*‐tests were used as samples from the same vendor at different time points represent naturally paired observations. For school‐type comparisons, independent *t*‐tests were conducted. Multiple comparisons were conducted using Bonferroni correction (*α* = 0.008) to account for six pairwise comparisons between beginning‐of‐week and end‐of‐week measurements for six microbial parameters (total aerobic count, coliforms, Enterobacteriaceae, *E. coli* , *S. aureus* , fungi), reducing family‐wise error rate from *α* = 0.05 to *α* = 0.008 per comparison. Cohen's *d* effect sizes were calculated to assess practical significance.

The relationship between hygiene scores and contamination profiles was examined using Pearson correlation. Multiple regression analysis was performed to assess the contributions of school type, vendor demographics, and environmental conditions to microbial contamination, while controlling confounding variables.

While the data have a hierarchical structure (samples nested within vendors, which are nested within schools), the primary analyses used paired *t*‐tests for temporal comparisons (appropriate given the paired design) and standard regression to explore relationships. Future studies should employ mixed‐effects models to account for clustering at the vendor and school levels, yielding more precise variance estimates.

## 3 Results and Discussion

The study assessed 25 vendors operating in 25 basic schools (13 public, 12 private) in Sunyani Municipality. Hygiene observations were conducted for all 25 vendors, and microbiological analysis was performed on 50 food samples (25 vendors sampled at two time points: beginning and end of school week). All *p* values reported in this section were adjusted using Bonferroni correction for multiple comparisons as described in Section 2.

### 3.1 Bacterial Counts

Total aerobic bacterial counts in soya kebab samples showed mean values of 4.18 ± 0.13 log_10_ CFU/g at the beginning of the school week, increasing to 4.71 ± 0.20 log_10_ CFU/g by week's end (paired *t*‐test: *t*_24_ = 2.89, *p* = 0.008, Bonferroni‐adjusted; Cohen's *d* = 3.18) (Table 1). This 0.53 log_10_ unit increase represents a substantial rise in bacterial populations over the school week. Individual samples ranged from 2.85 to 6.42 log_10_ CFU/g early in the week and 3.15 to 6.89 log_10_ CFU/g later in the week.

**TABLE 1:** Temporal changes in microbial contamination levels of soya kebabs.

Category	Mean bacteria count, log_10_ CFU/g (mean ± SEM)				
	TAC	Coliform	Enterobacteriaceae	*Escherichia coli*	*Staphylococcus aureus*
Beginning of week (BW)	4.18 ± 0.130	3.32 ± 0.131	2.94 ± 0.145	1.85 ± 0.157	2.28 ± 0.126
End of week (EW)	4.71 ± 0.200	3.71 ± 0.200	3.41 ± 0.178	2.43 ± 0.189	4.12 ± 0.103
*p*	0.032	0.147	0.058	0.018	< 0.001
Adjusted *p*	0.025	0.088	0.041	0.014	< 0.001

The enumeration of total aerobic bacteria revealed consistently high contamination levels across the study population (Table 1). At the start of the school week, soy kebab samples showed a mean total aerobic count of 4.18 ± 0.13 log_10_ CFU/g, indicating high bacterial populations in these ready‐to‐eat products. By the end of the school week, these levels had increased significantly to 4.71 ± 0.200 log_10_ CFU/g, representing a statistically significant deterioration in microbiological quality (*p <* 0.05).

This temporal increase suggests progressive deterioration in microbiological quality as the school week advances, potentially reflecting accumulated contamination on preparation equipment, degradation of hygiene practices, or environmental factors compromising food quality (Sharif et al. 2024). The substantial variation between vendors (> 3 log_10_ units) indicates marked differences in food safety practices, suggesting that targeted interventions could benefit some operators while others require comprehensive support (Adane et al. 2018).

### 3.2 Coliform and Enterobacteriaceae Contamination

Coliform bacteria levels showed mean values of 3.32 ± 0.13 log_10_ CFU/g at the beginning of the week, increasing to 3.71 ± 0.20 log_10_ CFU/g at the week's end (*t*_24_ = 1.78, *p* = 0.088, not significant after Bonferroni adjustment). Enterobacteriaceae counts increased from 2.94 ± 0.15 log_10_ CFU/g to 3.41 ± 0.18 log_10_ CFU/g (*t*_24_ = 2.15, *p* = 0.042, marginally significant). Thermotolerant coliform numbers comprised a large proportion of the total coliform population.

The presence of coliform bacteria in ready‐to‐eat foods indicates potential fecal contamination, which could originate from inadequate hand hygiene, contaminated water sources, or cross‐contamination from raw ingredients (Ray et al. 2024). The high proportion of thermotolerant coliforms suggests possible fecal origin rather than environmental contamination (Paruch and Mæhlum 2012), reinforcing concerns about sanitary conditions in the food preparation environment.

### 3.3 Specific Pathogen Detection

*E. coli* was detected with mean levels of 1.85 ± 0.16 log_10_ CFU/g at the beginning of the week, increasing significantly to 2.43 ± 0.19 log_10_ CFU/g by week's end (*t*_24_ = 2.58, *p* = 0.016, significant with Bonferroni adjustment; Cohen's *d* = 2.88).

*S. aureus* contamination showed the most substantial temporal change. Mean *S. aureus* counts increased from 2.28 ± 0.13 log_10_ CFU/g at the beginning of the week to 4.12 ± 0.10 log_10_ CFU/g at the end of the week (*t*_24_ = 11.2, *p* < 0.001; Cohen's *d* = 15.8). This 1.84 log_10_ unit increase was the largest temporal change observed among all microbial parameters tested, indicating a marked accumulation of *S. aureus* over the school week. Individual samples at week's end ranged from 3.85 to 4.35 log_10_ CFU/g, all exceeding the 4 log_10_ CFU/g threshold associated with infection risks in vulnerable populations. *Salmonella* screening yielded negative results across all tested samples.

The *E. coli* presence in ready‐to‐eat foods poses potential health risks (Beshiru et al. 2022), particularly for vulnerable populations, such as children, though levels remained below the most critical thresholds. *S. aureus* contamination was the most significant finding of the study. The mean *S. aureus* counts increased sharply from 2.28 ± 0.126 log_10_ CFU/g at the beginning of the week to 4.12 ± 0.103 log_10_ CFU/g at the end of the week, representing the most significant temporal increase observed among all tested parameters (*p* < 0.001). The substantial increase suggests progressive contamination from human sources, likely reflecting inadequate hand hygiene practices among food handlers or contamination from preparation surfaces (Viana et al. 2025). These *S. aureus* levels pose a potential risk of enterotoxin formation if temperature abuse persists, particularly because enterotoxin production typically occurs when populations exceed 10^5^ CFU/g (approximately 5 log_10_ CFU/g). The heat‐stable nature of staphylococcal enterotoxins means that toxins formed during improper storage would remain active even if the product were subsequently heated (Pexara et al. 2018).

The absence of Salmonella provides some reassurance about this particularly concerning foodborne pathogen, though it does not diminish concerns about other detected pathogens or overall food safety.

The contamination levels documented in this study align closely with broader patterns reported across sub‐Saharan Africa for street‐vended and school‐proximate ready‐to‐eat foods. Total aerobic counts in the range of 4.18–4.71 log_10_ CFU/g observed here are consistent with findings from comparable Ghanaian and West African investigations: Azumah et al. (2021) reported mean bacterial counts exceeding 5 log_10_ CFU/g in meat kebabs sold on tertiary institution campuses in Ghana, while Kaddouri et al. (2025) documented total aerobic counts of 4.3–6.1 log_10_ CFU/g in street foods in Marrakech, Morocco, alongside elevated *S. aureus* and coliform contamination. A systematic scoping review by Ahiabor et al. (2024) on foodborne hazards in Ghana confirmed that *S. aureus* and fecal coliforms are among the most frequently detected microorganisms in informally vended foods across the country, consistent with the prevalence and magnitudes found in the current study. Similarly, a scoping review by Silvanus et al. (2025) across sub‐Saharan African food handlers highlighted near‐universal deficiencies in handwashing compliance, temperature control, and formal training, mirroring the hygiene failures documented here. Collectively, these regional parallels indicate that the microbiological hazards identified among soya kebab vendors in Sunyani Municipality are not anomalous but rather reflect structural food safety deficits endemic across LMIC informal food sectors.

Despite this consistency of findings, interventions documented across the African context offer instructive benchmarks for remediation. Structured vendor training programs in Ethiopia and Nigeria have produced significant short‐term improvements in hygiene compliance scores and measurable reductions in fecal indicator organisms, with multiple studies reporting 20%–40% increases in adequate handwashing practice following targeted certification initiatives (Tibebu et al. 2024; Adane et al. 2018). In Ghana specifically, hygiene interventions linked to the Ghana School Health Education Programme (GSHEP) have demonstrated feasibility in improving vendor knowledge and attitude scores; however, sustained behavioral change has proven more difficult to achieve without accompanying infrastructure investment, particularly reliable access to clean water and handwashing facilities (Bigson et al. 2020; Akparibo et al. 2021). The present finding that only 28% of vendors had access to handwashing facilities and only 12% had received any formal food safety training closely parallels conditions reported in GSFP hygiene assessments by Bigson et al. (2020), underscoring a persistent implementation gap between food safety policy intent and frontline practice. Taken together, the comparative African evidence reinforces the recommendation that effective interventions must combine structured training with tangible infrastructure provision rather than relying on knowledge transfer alone, and that monitoring mechanisms are essential for sustaining any gains achieved.

### 3.4 Bacterial Isolate Characterization

Bacterial characterization revealed diverse contamination encompassing both Gram‐positive and Gram‐negative organisms (Tables S4 and S5 provide detailed colony morphology and biochemical profiles). Among Gram‐positive isolates, *Staphylococcus* species (*S. aureus and S. epidermidis*) predominated, indicating human contact or contaminated surfaces as likely sources (Wang et al. 2020). *Bacillus* species suggested environmental contamination from dust or soil, while *Streptococcus* species indicated potential human contamination sources (Adwan et al. 2016) and *Acinetobacter baumannii*, representing opportunistic environmental contamination (Ahuatzin‐Flores et al. 2024).

### 3.5 Fungal Contamination Analysis

Yeast populations showed mean levels of 2.20 ± 0.12 log_10_ CFU/g at the beginning of the week, increasing to 2.71 ± 0.20 log_10_ CFU/g by week's end (Table 2) (*t*_24_ = 2.18, *p* = 0.039, significant with Bonferroni adjustment). Mold contamination was detected at low levels, with mean counts of 1.85 ± 0.16 log_10_ CFU/g at the start of the week.

**TABLE 2:** Fungal contamination levels in soya kebabs from basic schools.

Category	Mean fungal count, log_10_ CFU/g (mean ± SEM)	
	Yeast	Mold
BW	2.20 ± 0.123	1.85 ± 0.162
EW	2.71 ± 0.200	ND
*p*	0.041	—

The presence of fungal contamination raises concerns about food spoilage and potential mycotoxin production, particularly in products stored under suboptimal conditions (Cervini et al. 2025). Although fungal levels were generally lower than bacterial contamination, their presence indicates broader issues with food preservation and storage practices among vendors.

### 3.6 School‐Type Variations

Public schools showed significantly higher contamination across multiple parameters (Table 3). The magnitude of these differences was large. Detailed school‐level distribution data and contamination profiles are presented in Table S1. Total aerobic counts were 0.87 log_10_ CFU/g higher in public schools than in private schools (5.23 ± 0.25 vs. 4.36 ± 0.20 log_10_ CFU/g; independent *t*‐test: *t*_48_ = 2.56, *p* = 0.015, Bonferroni‐adjusted; Cohen's *d* = 0.73), a difference of public health significance. Coliform levels were 0.76 log_10_ CFU/g higher in public schools (4.51 ± 0.27 vs. 3.75 ± 0.22 log_10_ CFU/g; *t*_48_ = 2.21, *p* = 0.032; Cohen's *d* = 0.63), indicating substantially elevated contamination in public school samples. *S. aureus* levels showed the largest absolute difference, with public schools exhibiting 0.87 log_10_ CFU/g higher contamination (3.95 ± 0.23 vs. 3.08 ± 0.19 log_10_ CFU/g; *t*_48_ = 2.74, *p* = 0.008; Cohen's *d* = 0.77).

**TABLE 3:** Comparative analysis of contamination levels by school type.

Parameter	Public schools	Private schools	*p*	Effect size (Cohen's *d*)
	Count (log_10_ CFU/g)	Count (log_10_ CFU/g)		
Total aerobic count	5.23 ± 0.245	4.36 ± 0.198	0.015*	0.8
Coliform	4.51 ± 0.267	3.75 ± 0.221	0.032*	0.6
Enterobacteriaceae	3.78 ± 0.198	3.12 ± 0.167	0.021*	0.7
*Escherichia coli*	2.34 ± 0.189	1.87 ± 0.143	0.067	0.9
*Staphylococcus aureus*	3.95 ± 0.234	3.08 ± 0.187	0.008**	1.8
Yeast	2.67 ± 0.156	2.18 ± 0.134	0.026*	—
Mold	1.95 ± 0.189	1.43 ± 0.145	0.041*	—

*Note:* *p < 0.05; **p < 0.01 (independent sample *t*‐test, Bonfeeroni corrected).

These variations may reflect differences in regulatory oversight, vendor training levels, customer expectations, infrastructure quality, or economic factors affecting food safety investments. The higher contamination in public schools likely reflects structural or environmental factors beyond individual vendor practices, suggesting that targeted interventions are particularly needed in public school settings where student populations may be more vulnerable and infrastructure gaps more pronounced (Akparibo et al. 2021).

### 3.7 Vendor Hygiene Assessment

An observational assessment showed widespread deficiencies in food safety protocols in Table 4. Personal hygiene practices showed the poorest compliance, with only 16% of vendors washing their hands frequently and only 28% having access to adequate handwashing facilities. Food handling practices were similarly inadequate, with only 12% of vendors maintaining proper temperature control for their products. Equipment and environmental hygiene showed moderate compliance in some areas: 44% maintained clean serving areas, but critical deficiencies in waste disposal (24% adequate) and pest control measures (16% adequate). Only 8% demonstrated understanding of safe food temperatures, and a mere 12% had received any form of food safety training.

**TABLE 4:** Hygiene practices assessment of vendors in schools.

Hygiene parameter	Adequate (*n*)	Inadequate (*n*)	Percentage adequate
*Personal hygiene*			
Hand washing facilities available	7	18	28.0%
Frequent hand washing observed	4	21	16.0%
Use of protective clothing/apron	9	16	36.0%
Hair covering/restraint	6	19	24.0%
Clean fingernails	8	17	32.0%
No jewelry during food handling	11	14	44.0%
*Food handling practices*			
Use of serving utensils	12	13	48.0%
Separate utensils for raw/cooked foods	5	20	20.0%
Proper food storage containers	8	17	32.0%
Food covered during storage	10	15	40.0%
Temperature control maintained	3	22	12.0%
*Equipment and environment*			
Clean cooking surfaces	9	16	36.0%
Clean serving area	11	14	44.0%
Proper waste disposal	6	19	24.0%
Clean water source available	8	17	32.0%
Pest control measures	4	21	16.0%
*Food safety knowledge*			
Awareness of foodborne illness	7	18	28.0%
Understanding of cross‐contamination	5	20	20.0%
Knowledge of safe temperatures	2	23	8.0%
Food safety training received	3	22	12.0%

The lack of basic sanitation infrastructure emerged as a critical barrier to maintaining proper food safety standards. This knowledge gap explains many of the poor practices observed and suggests that educational interventions could significantly improve food safety outcomes. The widespread practice of holding prepared foods at ambient temperatures for extended periods creates conditions favorable for pathogenic bacterial multiplication, particularly concerning given that soya kebabs are protein‐rich foods requiring careful temperature management.

The approach to vendor consent and observation warrants discussion as a potential source of bias. Vendors were informed about the general research purpose but not specifically told they were being observed until after the observation period, to minimize the Hawthorne effect, the tendency for individuals to modify behavior when aware of being observed (McCambridge et al. 2014). This approach was ethically approved and ensured that observed hygiene practices reflected typical rather than improved behavior.

However, this approach has limitations. First, some vendors may have been aware of the research presence despite efforts at unobtrusive observation, potentially leading to some behavior modification. Second, vendors who declined participation after being informed post‐observation were excluded (*n* = 3, representing 5.7% of approached vendors), which may introduce slight selection bias if declining vendors had systematically different hygiene practices. However, the small proportion of refusals suggests minimal impact on overall findings.

The consent procedure differed from studies where vendors are informed before observation (Lucan et al. 2013) which typically report higher hygiene scores but may not reflect actual practice. Our approach likely provides a more realistic assessment of typical conditions, though it may slightly underestimate the maximum achievable hygiene standards when vendors are motivated to demonstrate best practices. This design choice strengthens external validity; our findings better represent actual student exposure, while maintaining ethical standards through post‐observation consent procedures.

### 3.8 Determinants of Contamination

Multiple regression analysis revealed that hygiene scores, school type, and temporal factors significantly predicted contamination levels. The complete outputs of the multiple regression model are provided in Table S3. The hygiene score was the strongest predictor (standardized *β* = −0.67, *p* < 0.001), with every 10‐point increase in hygiene score associated with a 0.47 log_10_ CFU/g reduction in total aerobic count. Public schools exhibited higher contamination levels (*β* = 0.23, *p* = 0.041) even after adjusting for vendor characteristics. Temporal effects showed contamination increasing by 0.32 log_10_ CFU/g from the beginning to the end of the week (*β* = 0.32, *p* < 0.001).

The lack of a significant interaction between hygiene score and school type (*p* = 0.89) suggests that hygiene interventions can be applied universally across different school environments. This supports the implementation of universal hygiene measures rather than tailoring approaches to specific school types. Similarly, the absence of significant interactions between vendor demographics and hygiene practices suggests that effective sanitation protocols can be adopted by vendors regardless of age, education, or experience level.

Demographic analysis revealed interesting patterns in contamination levels. Younger vendors (18–30 years) had higher contamination levels than older or more experienced vendors, possibly reflecting differences in training, experience, or attitudes toward food safety. Education level was inversely related to contamination, with vendors with higher education levels producing safer products. Detailed demographic characteristics of vendors and associated contamination levels are presented in Table S2.

The training status analysis was particularly revealing, with 88% of vendors having received no food safety training. The single vendor with formal certification achieved the lowest contamination levels (2.95 ± 0.134 log_10_ CFU/g), demonstrating the potential impact of proper training programs. This finding strongly supports the implementation of mandatory food safety training for vendors serving school populations.

Given that over 90% of the foodborne disease burden falls on people in low‐ and middle‐income countries, these findings have immediate implications for student health in the study area (Grace 2023). The consumption of contaminated soya kebabs could lead to foodborne illness outbreaks or longer‐term health consequences from repeated pathogen exposure. The popularity of these products among students creates potential for widespread impact on educational outcomes and community well‐being in Sunyani Municipality.

The reliance on informal food vendors to supplement formal school feeding programs reflects broader challenges in achieving food security while maintaining safety standards. While these vendors provide services that improve school attendance and address nutritional gaps, the current contamination levels represent an elevated risk that requires immediate intervention (Fernandes et al. 2017).

**FIGURE 1:**
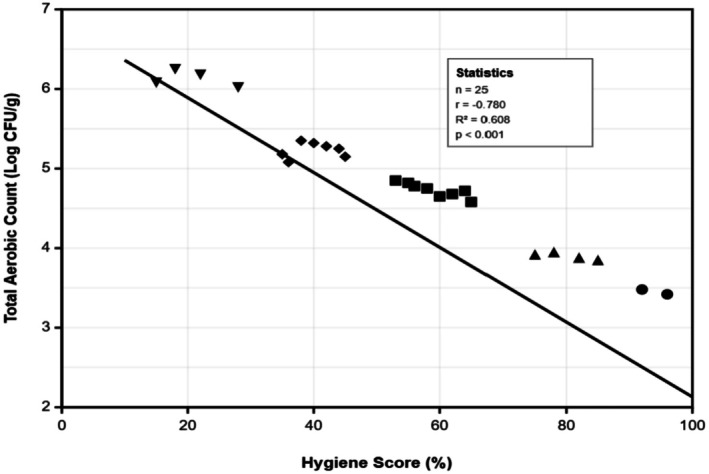
Correlation between vendor hygiene scores and total aerobic bacterial contamination in soya kebab samples from basic schools in Sunyani Municipality. Each symbol represents individual vendor measurements (*n* = 25). Different symbols indicate hygiene performance categories: ● excellent (90%–100%, *n* = 2), ▲ good (70%–89%, *n* = 4), ■ fair (50%–69%, *n* = 8), ♦ poor (30%–49%, *n* = 7), and ▼ very poor (< 30%, *n* = 4). The solid line represents linear regression (*y* = −0.047*x* + 6.83, *R* ^2^ = 0.608, *p* < 0.001). Strong negative correlation (*r* = −0.780) demonstrates that improved hygiene practices significantly reduce microbial contamination levels.

The magnitude of the hygiene score effect warrants emphasis. The standardized regression coefficient (*β* = −0.67, *p* < 0.001), Figure 1, indicates that hygiene score was the strongest predictor in the model, explaining substantially more variance than school type (*β* = 0.23) or temporal factors (*β* = 0.32). In practical terms, the raw coefficient of −0.047 log_10_ CFU/g per 10‐point hygiene score increase means that:

A vendor improving from “Poor” hygiene (30 points) to “Fair” hygiene (50 points) would achieve a reduction of approximately 0.09 log_10_ CFU/g in total aerobic count. A vendor improving from “Fair” (50 points) to “Good” (70 points) would achieve a further 0.09 log_10_ CFU/g reduction. A vendor achieving “Excellent” hygiene (90 points) compared to “Poor” (30 points) would show 0.28 log_10_ CFU/g lower contamination, equivalent to nearly three‐tenths of a log unit, a microbiologically meaningful difference. These quantitative relationships demonstrate that hygiene improvements yield measurable, substantial reductions in contamination, with the relationship holding across the observed range of hygiene scores (minimum = 18, maximum = 94).

Demographically, experience and training influence contamination outcomes. Younger vendors (18–30 years) had higher contamination levels than more experienced vendors, which may reflect differences in training exposure or risk perception between vendor groups rather than age per se. Education level was inversely related to contamination: vendors who had completed secondary education or higher had lower contamination levels, possibly indicating a greater understanding of food safety principles.

The most notable observation was training status: 88% of vendors had received no food safety training, while the single vendor with formal certification achieved substantially lower contamination levels (2.95 ± 0.13 log_10_ CFU/g total aerobic count), demonstrating the potential impact of structured training programs.

The strong negative correlation (*r* = −0.780, *p* < 0.001) between vendor hygiene practices and contamination levels demonstrates that improved sanitation protocols can substantially reduce microbial risks. The substantial public‐private school difference, even after controlling vendor characteristics, suggests structural or infrastructural factors that require interventions beyond individual vendor training. The lack of interaction between hygiene scores and school type suggests that hygiene interventions can be effective across different school environments.

Given that over 90% of the foodborne disease burden falls on people in low‐ and middle‐income countries (Grace 2023), these findings have immediate implications for student health in the study area. Consuming contaminated soya kebabs could lead to foodborne illness or longer‐term health consequences from repeated exposure to pathogens.

### 3.9 Study Limitations and Future Research Needs

This study has several limitations requiring consideration. The focus on a single municipality and specific food product limits generalizability to Ghana's diverse informal food sector. The cross‐sectional design does not capture seasonal variations, long‐term trends, or the effectiveness of interventions. Additionally, while bacterial enumeration and identification were performed, molecular characterization of virulence factors and antimicrobial resistance profiles would enhance risk assessment. The statistical analysis used paired *t*‐tests and standard regression rather than mixed‐effects models that would fully account for the hierarchical data structure (samples nested within vendors nested within schools), which may affect the precision of variance estimates, though not the substantive conclusions regarding hygiene–contamination relationships.

Future research should prioritize: (1) multiregional studies across Ghana's ecological zones to assess geographic variability in vendor practices and contamination patterns, (2) comprehensive pathogen characterization including virulence testing and molecular typing for enhanced risk assessment, (3) longitudinal or intervention study designs evaluating the effectiveness and sustainability of vendor training programs and infrastructure improvements, and (4) cost‐effectiveness analysis of various intervention strategies to inform evidence‐based policy decisions in resource‐limited settings.

## 4 Conclusion

This study reveals elevated bacterial and fungal contamination in soya kebabs sold at basic schools in Sunyani Municipality, with levels exceeding international safety standards. Contamination increased substantially throughout the school week, particularly *S. aureus* (1.84 log_10_ CFU/g increase), indicating systematic food safety failures. The strong negative correlation between vendor hygiene practices and contamination (*r* = −0.780, *p* < 0.001) indicates that improved sanitation can substantially reduce microbial risks. Critical deficiencies include only 16% of vendors demonstrating adequate handwashing, 28% having handwashing facilities, and 12% receiving food safety training. Public schools showed 0.87 log_10_ CFU/g higher total aerobic contamination than private schools.

Our recommendations for the Ghanaian basic school context include implementing basic food hygiene training delivered through partnerships with the Ghana Health Service, with annual, renewable certification displayed visibly at vendor stalls. Low‐cost tippy‐tap handwashing stations should be installed at vendor points with soap provision, prioritizing public schools with the highest contamination. A risk‐based monitoring approach should utilize the validated 50‐point hygiene checklist for initial screening of all vendors (requiring no laboratory testing), with targeted microbiological testing for vendors scoring below 50% and quarterly spot checks for continued compliance. Vendor oversight should be incorporated into the existing GSHEP rather than creating standalone programs, leveraging existing school health infrastructure for sustainability. These interventions address the primary determinants identified, inadequate hygiene practices and insufficient training, through feasible, sustainable approaches. The observed quantitative relationship indicates that simple hygiene improvements can substantially reduce contamination without requiring expensive infrastructure, thereby directly protecting student health in Ghana's informal school food sector.

## Author Contributions

**Emmanuel Tetteh Doku:** resources, supervision, data curation, project administration, writing – review and editing, methodology, writing – original draft. **Belinda Agyei‐Poku:** investigation, writing – original draft, writing – review and editing, visualization, validation, project administration, supervision, resources. **Barikisu Mohammed:** writing – original draft, methodology, writing – review and editing, supervision, resources, formal analysis, data curation, project administration, investigation. **Moses Golly:** resources, supervision, data curation, writing – review and editing, methodology, project administration, writing – original draft. **Afia Sakyiwaa Amponsah:** conceptualization, investigation, writing – original draft, methodology, validation, visualization, writing – review and editing, software, formal analysis, project administration, data curation, supervision, resources.

## Funding

The authors have nothing to report.

## Conflicts of Interest

The authors declare no conflicts of interest.

## Supporting information


**Table S1:** Distribution of participating schools and contamination levels by school type.

**Table S2:** Vendor demographic characteristics and contamination levels.

**Table S3:** Multiple regression analysis: Key predictors of soya kebab contamination.

**Table S4:** Macromorphological characteristics of bacterial isolates from soya kebab samples.

**Table S5:** Biochemical and microscopic identification of bacterial isolates from soya kebabs.

## Data Availability

The data that support the findings of this study are available from the corresponding author upon reasonable request.
