# Supplementary material for: Vendor Hygiene Practices, Temporal Variation, and Microbial Quality of Soya Kebabs Sold in Public and Private Basic Schools in Sunyani, Ghana
Source: Food Sci Nutr. 2026 May 23;14(5):e71909. doi: 10.1002/fsn3.71909 (PMC13239884; doi:10.1002/fsn3.71909)
Supplement: Supplementary file 1 — Table S1: Distribution of participating schools and contamination levels by school type. [file FSN3-14-e71909-s005.docx]

**Supplementary Table 1. Distribution of participating schools and contamination levels by school type**

| **School Name** | **School Type** | **Location** | **Vendor Status** | **Mean TAC (log₁₀ CFU/g)** | **Mean Coliform (log₁₀ CFU/g)** | **Mean S. aureus (log₁₀ CFU/g)** |
| --- | --- | --- | --- | --- | --- | --- |
| Abesim Presby A | Public | Abesim | On-campus | 5.85 | 4.21 | 3.45 |
| Olister Edu. Complex | Private | Town Center | Near-campus | NBG | NBG | NBG |
| Adventist Preparatory | Private | Bono Road | On-campus | 4.12 | 3.64 | 2.85 |
| Barhamiya Islamic School | Public | Muslim Quarter | On-campus | 3.45 | 2.87 | 1.95 |
| Estate Experimental | Public | New Estate | Near-campus | NBG | NBG | NBG |
| High Street School | Public | High Street | Near-campus | NBG | NBG | NBG |
| All Saints R/C School | Public | Cathedral Area | On-campus | NBG | NBG | NBG |
| St. Anslem Anglican | Private | Anglican Mission | On-campus | NBG | NBG | NBG |
| AME Zion School | Public | Zion Hill | On-campus | 4.68 | 3.95 | 3.12 |
| Susec Model | Private | Susec Estate | On-campus | 3.89 | 3.24 | 2.76 |
| St. James/St Patrick R/C | Public | Catholic Mission | On-campus | 6.14 | 5.38 | 4.85 |
| Mwawasua MA Basic | Public | Mwawasua | Near-campus | NBG | NBG | NBG |
| South Ridge Model | Private | South Ridge | On-campus | 3.67 | 3.15 | 2.42 |
| Abesim Presby B | Public | Abesim Junction | On-campus | 4.92 | 4.18 | 3.67 |
| Sacred Heart School | Private | Sacred Heart | On-campus | 5.74 | 4.96 | 4.23 |
| PA Capital School | Private | Capital Estate | On-campus | 4.35 | 3.78 | 3.08 |
| Nyamaa Basic School | Public | Nyamaa | On-campus | 6.28 | 5.65 | 5.14 |
| Abesim Methodist | Public | Methodist Mission | On-campus | 5.15 | 4.47 | 3.89 |
| Presby School | Public | Presbyterian | On-campus | 4.56 | 3.98 | 3.21 |
| Islamic School | Public | Central Mosque | On-campus | NBG | NBG | NBG |
| Ridge Experimental | Public | Ridge Area | Near-campus | 5.89 | 5.12 | 4.56 |
| MA Basic School | Public | Municipal Assembly | Near-campus | NBG | NBG | NBG |
| Ghana Muslim Mission | Public | Muslim Mission | On-campus | 5.67 | 4.89 | 4.12 |
| Wise Education Complex | Private | Wise Junction | On-campus | 4.78 | 4.23 | 3.54 |
| Community Basic School | Public | Community Center | Near-campus | NBG | NBG | NBG |
