# Supplementary material for: Vendor Hygiene Practices, Temporal Variation, and Microbial Quality of Soya Kebabs Sold in Public and Private Basic Schools in Sunyani, Ghana
Source: Food Sci Nutr. 2026 May 23;14(5):e71909. doi: 10.1002/fsn3.71909 (PMC13239884; doi:10.1002/fsn3.71909)
Supplement: Supplementary file 2 — Table S2: Vendor demographic characteristics and contamination levels. [file FSN3-14-e71909-s001.docx]

Supplementary Table 2. Vendor demographic characteristics and contamination levels

| **Parameter** | **Category** | **Number (n)** | **Percentage (%)** | **Mean Contamination Level** |
| --- | --- | --- | --- | --- |
| **Age Group** |  |  |  | **(TAC log₁₀ CFU/g)** |
|  | 18-30 years | 12 | 48.0% | 4.89 ± 0.234 |
|  | 31-45 years | 8 | 32.0% | 4.45 ± 0.198 |
|  | 46-60 years | 5 | 20.0% | 4.12 ± 0.167 |
| **Education Level** |  |  |  |  |
|  | No formal education | 6 | 24.0% | 5.34 ± 0.289 |
|  | Primary education | 11 | 44.0% | 4.78 ± 0.245 |
|  | Junior High School | 7 | 28.0% | 4.23 ± 0.198 |
|  | Senior High School | 1 | 4.0% | 3.45 ± 0.156 |
| **Years of Experience** |  |  |  |  |
|  | <1 year | 8 | 32.0% | 5.12 ± 0.267 |
|  | 1-3 years | 9 | 36.0% | 4.67 ± 0.234 |
|  | 3-5 years | 6 | 24.0% | 4.34 ± 0.189 |
|  | >5 years | 2 | 8.0% | 3.89 ± 0.145 |
| **Training Status** |  |  |  |  |
|  | No food safety training | 22 | 88.0% | 4.89 ± 0.245 |
|  | Informal training | 2 | 8.0% | 3.78 ± 0.167 |
|  | Formal certification | 1 | 4.0% | 2.95 ± 0.134 |
