# Supplementary material for: Vendor Hygiene Practices, Temporal Variation, and Microbial Quality of Soya Kebabs Sold in Public and Private Basic Schools in Sunyani, Ghana
Source: Food Sci Nutr. 2026 May 23;14(5):e71909. doi: 10.1002/fsn3.71909 (PMC13239884; doi:10.1002/fsn3.71909)
Supplement: Supplementary file 3 — Table S3: Multiple regression analysis: Key predictors of soya kebab contamination. [file FSN3-14-e71909-s003.docx]

**Supplementary Table 3. Multiple regression analysis: Key predictors of soya kebab contamination**

| **Predictor** | **β Coefficient** | **Standardized β** | **P value** | **95% CI** |
| --- | --- | --- | --- | --- |
| **Hygiene Score (%)** | **-0.047** | **-0.67** | **<0.001** | **-0.063 to -0.031** |
| **School Type (Public)** | **0.23** | **0.31** | **0.041** | **0.01 to 0.45** |
| **Vendor Education** | **-0.15** | **-0.18** | **0.068** | **-0.31 to 0.01** |
| **Vendor Age** | **0.008** | **0.12** | **0.378** | **-0.010 to 0.026** |
| **Experience Years** | **-0.012** | **-0.08** | **0.607** | **-0.058 to 0.034** |
