# Supplementary material for: Vendor Hygiene Practices, Temporal Variation, and Microbial Quality of Soya Kebabs Sold in Public and Private Basic Schools in Sunyani, Ghana
Source: Food Sci Nutr. 2026 May 23;14(5):e71909. doi: 10.1002/fsn3.71909 (PMC13239884; doi:10.1002/fsn3.71909)
Supplement: Supplementary file 4 — Table S4: Macromorphological characteristics of bacterial isolates from soya kebab samples. [file FSN3-14-e71909-s004.docx]

**Supplementary Table 4. Macromorphological characteristics of bacterial isolates from soya kebab samples**

| **Opacity** | **Shape** | **Size** | **Colour** | **Margin** | **Elevation** |
| --- | --- | --- | --- | --- | --- |
| Opaque | Circular | Small | White | Even | Flat |
| 12 (75%) | 14 (88%) | 8 (50%) | 3 (19%) | 12 (75%) | 10 (63%) |
| Translucent | Irregular | Medium | Yellow | Undulate | Raised |
| 4 (25%) | 2 (12%) | 6 (38%) | 4 (25%) | 4 (25%) | 6 (37%) |
|  |  | Large | Cream |  |  |
|  |  | 2 (12%) | 9 (56%) |  |  |
| 16 (100%) | 16 (100%) | 16 (100%) | 16 (100%) | 16 (100%) | 16 (100%) |
