# Supplementary material for: Vendor Hygiene Practices, Temporal Variation, and Microbial Quality of Soya Kebabs Sold in Public and Private Basic Schools in Sunyani, Ghana
Source: Food Sci Nutr. 2026 May 23;14(5):e71909. doi: 10.1002/fsn3.71909 (PMC13239884; doi:10.1002/fsn3.71909)
Supplement: Supplementary file 5 — Table S5: Biochemical and microscopic identification of bacterial isolates from soya kebabs. [file FSN3-14-e71909-s002.docx]

**Supplementary Table 5. Biochemical and Microscopic Identification of Bacterial Isolates from Soya Kebabs**

| **Isolate** |  |  |  |  |  |  |  |  | **Microscopy** | |  | | |  |  |
| --- | --- | --- | --- | --- | --- | --- | --- | --- | --- | --- | --- | --- | --- | --- | --- |
|  | **T1** | **T2** | **T3** | **Glu** | **Lac** | **Suc** | **H₂S** | **Gas** | **Shape** | **Order** | | **Reaction** | | | **Inference** |
| SK1 | + | + | - | - | - | - | - | - | Rods | Singular | | | Positive | | *Bacillus* sp. |
| SK2 | + | + | + | - | - | - | - | - | Circular | Cluster | | | Positive | | *Staphylococcus* sp. |
| SK3 | + | + | + | + | - | - | - | - | Circular | Singular | | | Negative | | *Acinetobacter* sp. |
| SK4 | + | + | + | + | - | - | + | - | Circular | Cluster | | | Positive | | *Staphylococcus aureus* |
| SK5 | + | + | - | + | - | - | + | - | Circular | Singular | | | Negative | | *Enterobacter* sp. |
| SK6 | + | + | + | + | - | - | - | - | Circular | Cluster | | | Positive | | *Staphylococcus epidermidis* |
| SK7 | - | + | - | + | - | - | - | - | Circular | Chains | | | Positive | | *Streptococcus* sp. |
| SK8 | + | + | + | + | - | - | - | - | Rods | Singular | | | Negative | | *Escherichia coli* |
| SK9 | + | + | + | + | - | - | - | - | Rods | Pairs | | | Negative | | *Klebsiella* sp. |
| SK10 | + | - | + | + | + | - | - | + | Rods | Singular | | | Negative | | *Enterobacter aerogenes* |

T1 – Catalase, T2 – Oxidase, T3 – Citrate, Glu – Glucose, Lac – Lactose, Suc – Sucrose, H₂S – Hydrogen sulphide
